# An FPGA-Based YOLOv5 Accelerator for Real-Time Industrial Vision Applications

**DOI:** 10.3390/mi15091164

**Published:** 2024-09-19

**Authors:** Zhihong Yan, Bingqian Zhang, Dong Wang

**Affiliations:** 1 Institute of Information Science, Beijing Jiaotong University, Beijing 100044, China; 23115053@bjtu.edu.cn (Z.Y.); 22120334@bjtu.edu.cn (B.Z.); 2 Beijing Key Laboratory of Advanced Information Science and Network Technology, Beijing 100044, China

**Keywords:** FPGA accelerator, YOLO, 4bit, quantization, object detection

## Abstract

The You Only Look Once (YOLO) object detection network has garnered widespread adoption in various industries, owing to its superior inference speed and robust detection capabilities. This model has proven invaluable in automating production processes such as material processing, machining, and quality inspection. However, as market competition intensifies, there is a constant demand for higher detection speed and accuracy. Current FPGA accelerators based on 8-bit quantization have struggled to meet these increasingly stringent performance requirements. In response, we present a novel 4-bit quantization-based neural network accelerator for the YOLOv5 model, designed to enhance real-time processing capabilities while maintaining high detection accuracy. To achieve effective model compression, we introduce an optimized quantization scheme that reduces the bit-width of the entire YOLO network—including the first layer—to 4 bits, with only a 1.5% degradation in mean Average Precision (mAP). For the hardware implementation, we propose a unified Digital Signal Processor (DSP) packing scheme, coupled with a novel parity adder tree architecture that accommodates the proposed quantization strategies. This approach efficiently reduces on-chip DSP utilization by 50%, offering a significant improvement in performance and resource efficiency. Experimental results show that the industrial object detection system based on the proposed FPGA accelerator achieves a throughput of 808.6 GOPS and an efficiency of 0.49 GOPS/DSP for YOLOv5s on the ZCU102 board, which is 29% higher than a commercial FPGA accelerator design (Xilinx’s Vitis AI).

## 1. Introduction

Deep learning models have been widely applied in various fields of artificial intelligence. Among these models, the YOLO series has attracted widespread attention from the research and industrial sectors in the field of object detection due to its good balance between accuracy and computational cost [1]. In order to maintain the detection speed and low-cost inference of YOLO in industrial applications, the FPGA-based YOLO accelerator design has become a popular deployment solution [1,2,3,4].

To meet the demand for high inference speed, model quantization has become a widely adopted approach for performance optimization. Early quantization research was limited to 8–16 bits to ensure model precision [5,6,7]. However, 8–16-bit quantization consumes a significant amount of hardware resources, such as DSP and Look Up Table (LUT), when deployed on an FPGA device, thereby limiting performance gains. For instance, Montgomerie-Corcoran et al. [5] developed an FPGA accelerator using 8-bit weights and 16-bit activations. Despite efforts to efficiently utilize hardware resources, the design consumed 1794 DSPs and 602k LUTs, delivering only a throughput of 392 GOP/s on the VCU110 development board (Xilinx, CA, USA).

Consequently, recent research has focused on further compressing the bit width of YOLO accelerators. However, accelerating low-bit YOLO models still presents numerous challenges. When the quantization bit width becomes lower than 8 bits, the model experiences a significant accuracy drop [8]. Although some nonuniform and symmetric quantization methods perform well, their hardware resource consumption and the need for floating-point computation make them unsuitable for FPGA deployment [9,10,11].

So far, the low-bit (≤4-bit) YOLO accelerator designs face the requirements of high speed, low hardware resources, and good detection accuracy. To address the aforementioned issues, this paper proposes a hardware-friendly, full-network 4-bit quantization algorithm and an FPGA-based YOLO accelerator design, achieving high inference accuracy and throughput simultaneously. The contributions of this work are summarized as follows:

(1) Introduction of a hardware-friendly low-bit-width quantization scheme for the YOLO detection model. This novel scheme incorporates three key optimizations: first-layer quantization, activation function, and quantization function. For first-layer quantization, we propose Mimic 4-bit Quantization (M4bQ), which efficiently reduces 8-bit input pixel values to 4 bits without compromising accuracy. This technique eliminates the need for resource-intensive mixed-precision convolution circuits in the hardware accelerator. For the activation function, we introduce Offset PReLU (OPReLU), a flexible approach that reshapes input data to improve model accuracy. Finally, for the quantization function, we propose Signed Clipping Quantization (SCQ), which quantizes activations into 4 bits by employing two clipping points to select the optimal quantization range. This ensures a symmetric and uniform distribution, preserving high accuracy throughout the network.
(2) Design of an efficient accelerator for FPGA Deployment. To accommodate the proposed quantization scheme, we introduce an efficient DSP packing strategy and a flexible adder tree circuit design. Our DSP packing scheme enables four parallel 4-bit multiplications within a single DSP48e1/2 block, thereby reducing on-chip DSP utilization by 50% compared with conventional 8-bit packing schemes [7]. The adder tree architecture is designed to handle different input data formats produced by M4bQ and SCQ, maximizing resource sharing and further improving the overall efficiency of the hardware accelerator.
(3) Implementing a 4-bit FPGA accelerator for YOLO detectors on Xilinx’s ZCU102 board (Xilinx, CA, USA) and achieving an inference throughput of 808.6 GOP/s and 0.49 GOPS/DSP for the YOLOv5s model, improving the performance of a commercial FPGA accelerator (Xilinx’s Vitis AI) by 29% on the same device.

## 2. Related Works

Previous researchers have conducted some fruitful explorations on accelerators for YOLO networks. Valadanzoj et al. [12] proposed an accelerator with 5-bit weights and 8-bit activations for the YOLOv4-tiny, which resulted in a 3% accuracy drop and a DSP packing scheme that was as effective as pure 8-bit accelerators [7]. Zhang et al. [13] developed a quantization method for YOLOv3-tiny with 2-bit weights and 8-bit activations, achieving a 2% accuracy decrease and implementing a density of 0.44 GOPS/DSP on an FPGA. Javed et al. [14] quantized the YOLOv2-tiny based on 1-bit weights and 4-bit activations and deployed it on an FPGA using a scalable binary accelerator framework. While the accelerator demonstrated good energy and resource efficiency, it incurred an accuracy loss of 5.6% compared with the float-point model. Zhao et al. [15] proposed a 4-bit quantization method and designed a LUT-based FPGA accelerator, achieving a high hardware utilization of 6.7 GOPS/DSP on the YOLOv2 network. However, their proposed quantization method only maintained minimal accuracy degradation on the classification network and no experiments were reported on the YOLO network. Sommer et al. [16] promoted Xilinx’s DSP packaging [17] and explained the sources and correction methods of errors in the original scheme. More importantly, they proposed “Overpacking”, which compresses six 4-bit multiplications into one DSP at the cost of small errors. However, in many neural network scenarios, activations can also be signed, limiting the general applicability of this method. Furthermore, applying “Overpacking” during neural network inference could introduce additional accuracy degradation, which may be difficult to mitigate through other compensatory techniques.

In summary, existing low-bit (≤4-bit) YOLO accelerator designs exhibit two key limitations. First, there is a scarcity of quantization schemes that can simultaneously ensure hardware efficiency and high accuracy. Second, current accelerator designs fail to fully exploit the benefits of low-bit quantization to achieve both minimal hardware resource utilization and high throughput. To address these challenges, this work proposes several optimizations in quantization techniques and FPGA accelerator design.

## 3. Principle of the YOLOv5s Algorithm

**Figure 1:**
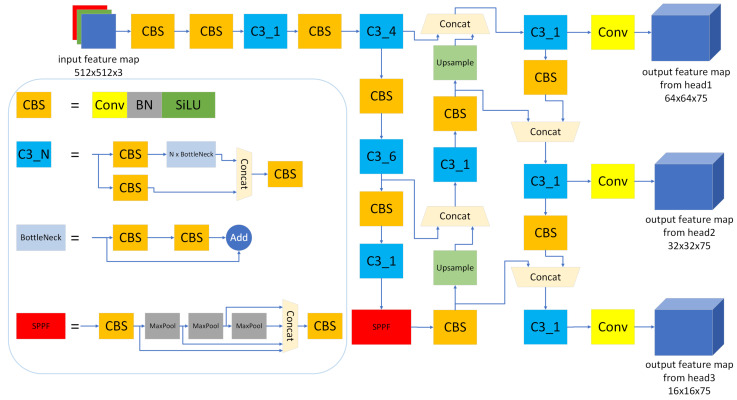
Structure of YOLOv5s. The CBS block consists of a continuous convolution layer, a BN layer, and a SiLU activation. The BottleNeck is a residual structure composed of multiple CBS blocks. The CBSx3_N (C3_N) module contains three CBS blocks, one concat layer, and *N* BottleNecks.

Since its inception, the YOLO series algorithms have received widespread attention for their excellent detection speed and reliable detection accuracy, becoming one of the representatives of one-stage detection networks. Among them, the YOLOv5 series [18] provide researchers and developers with rich model versions, supporting multiple balance points between inference speed and model accuracy. This paper evaluated the existing YOLO algorithms in terms of network accuracy and speed and, finally, chose YOLOv5s as the model that can meet the requirement of a Printed Circuit Board (PCB) defect detection application. The detailed network structure of the YOLOv5s model is shown in Figure 1.

YOLOv5s itself has undergone multiple network structure adjustments, and this paper uses the latest 6.0 version as the basic model for object detection networks. The basic modules of YOLOv5s are the convolution layer, the batch normalization (BN) layer, and the Sigmoid Linear Unit (SiLU) activation (CBS). Convolution layers are used to extract local spatial information from input features, typically consisting of multiple convolution kernels, each of which extracts a type of spatial information. The BN layer is used to standardize the activation distribution on each channel of the input feature-map. It can accelerate the training process and improve the model’s generalization ability while reducing the model’s dependence on initialization. SiLU is used as an activation function to introduce nonlinear transformation capability to neural networks. In addition, its smooth feature at zero points can facilitate better transmission of gradient information in the network [19]. In the quantization process, SiLU will be replaced with OPReLU proposed in this paper to improve quantization accuracy. During the inference process, the BN layer is fused with the convolution layer to reduce computational complexity.

On the basis of the CBS module, YOLOv5s constructs the CBSx3 (C3) structure, which integrates features from different levels and uses residual connections to provide better gradient transfer. Its main function is to increase the depth and receptive field of the network and improve the ability of feature extraction. The C3 module contains multiple Conv layers, where a stride of two convolutions can reduce the size of the feature-map by half, increase the receptive field of the network, and reduce computational complexity. By halving the size of the feature-map, the network can focus more on the global information of objects, thereby improving the effectiveness of feature extraction. Convolution with stride 1 does not change the size of the feature-map, which can maintain the spatial resolution of the feature-map and better preserve the local information of the object. In addition, YOLOv5s has improved the Spatial Pyramid Pooling (SPP) structure by proposing the Spatial Pyramid Pooling-Fast (SPPF) module, which achieves faster inference speed with less computation while still maintaining the functions of avoiding duplicate feature extraction and image distortion in the SPP module [20].

In terms of macrostructure, YOLOv5s still adopts the three-level structure of previous YOLO models, namely backbone, neck, and head. The backbone is responsible for feature extraction, the neck performs multiscale feature fusion through upsample and downsample operations, and the head completes the final regression detection.

## 4. Full-Network 4-Bit Quantization

Recent studies have proposed various schemes for 4-bit quantization [21,22]. Notably, asymmetric and nonuniform quantization schemes tend to achieve superior accuracy compared with their symmetric and uniformed counterparts [10]. However, the intrinsic complexity of asymmetric and nonuniform data formats poses significant challenges for hardware implementation. For example, in convolution computations, weights and activations quantized using nonuniform schemes must be converted back to floating-point numbers, which will inevitably consume substantial hardware resources and degrade the accelerator’s performance [11]. Conversely, the accuracy of symmetric and uniform quantization is often inferior to that of asymmetric and nonuniform methods [10]. Therefore, a hardware-friendly and high-accuracy quantization method is needed.

In this work, we proposed a symmetric and uniform quantization scheme that can deliver SOTA-level model accuracy, while ensuring efficiency in hardware implementation. The novel quantization scheme incorporates three specific optimizations: a 4-bit quantization for the first layer, an optimized activation function, and a quantization function.

### 4.1. OPReLU Function

In YOLOv5s the SiLU function is used as the activation function. However, the fixed slope on the negative axis of input *x* and the lack of flexibility in horizontal shifting impede its ability to effectively adapt to the distribution of input data. To address this issue, we introduce a trainable offset to the PReLU function [23] to solve the problem. The novel Offset PReLU (OPReLU) function is defined as Equation (1):

(1) $$OPReLU(x)=\left\{\begin{array}{cc} x-o, & \mathrm{if}\;x>o\\ w(x-o), & \mathrm{otherwise}. \end{array}\right.$$

where *x* denotes the function input, *w* represents the learnable slope, and *o* indicates the offset value. The adjustable offset *o* enables the activation function to shift along the x-axis, allowing for better alignment of the distribution of the feature-map with the quantization range in each layer during the quantization training process. This adjustment effectively minimizes the quantization error. For hardware implementation, the subtraction of the offset requires only an additional adder in the convolution computation circuit, resulting in no obvious increase in external memory access.

### 4.2. Quantization Function

In this work, activations are quantized into 4 bits by using the proposed Signed Clipping Quantization (SCQ) scheme, whereas weights are quantized using the Learned Step Size Quantization (LSQ) method [24]. As defined in Equation (2), SCQ is designed to clip values from both the positive and negative half-axes, thereby focusing on representing values in the data-intensive region.

(2) $$\begin{array}{c} q=clip(q_{1},-\left(2^{bit-1}\right),2^{bit-1}-1) \end{array}$$

(3) $$\begin{array}{c} q_{1}=round\left(clip\left(f,-c,c\right)/scale\right) \end{array}$$

(4) $$\begin{array}{c} scale=c/(2^{bit-1}) \end{array}$$

where *q* represents the quantization value, scale is the scaling ratio, and *c* denotes a learnable clipping parameter, which is constrained to a power of 2 value to minimize the hardware implementation cost. The variable bit indicates the data bit width, and *f* refers to the original floating-point value. The learnable clipping parameter *c* determines the optimal quantization interval, ensuring that the distribution range of the floating-point outputs of the convolution is adequately covered. This allows the maximum and minimum values to be accurately mapped to the corresponding fixed-point numbers.

### 4.3. First Layer Quantization

Previous studies have indicated that directly quantizing the 8-bit input of the first layer to lower bit widths, such as 4 or 2 bits, results in significant accuracy loss [9,25]. However, maintaining the 8-bit data width of the first layer necessitates that the hardware accelerator supports mixed-precision convolution computation, as other layers are quantized with low bit widths. Mixed-precision convolution circuits, however, are costly in terms of hardware implementation. To enhance resource efficiency, we introduce a novel quantization method called Mimic 4-bit Quantization (M4bQ), which quantizes the 8-bit input of the first layer into 4 bits without accuracy loss.

The core idea of M4bQ is to requantize each 8-bit input pixel value of the first layer into two 4-bit fixed-point numbers in such a manner that the convolution results remain unchanged. The M4bQ for the convolution calculation process is divided into three steps. M4bQ is based on 8-bit quantization of the first layer input, so the first step of M4bQ is to use SCQ to quantize the input of the first layer to 8 bits. Afterward, two 4-bit fixed-point numbers are generated using requantizing. Lastly, adjusted convolution is performed.

**Figure 2:**
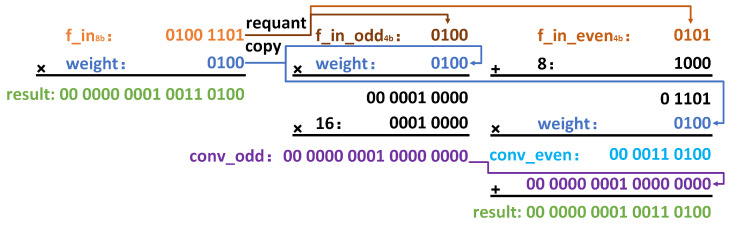
Requantizing from 8 bit to 4 bit.

Take an example of convolution between a single channel 1 × 1 input feature-map and a single-channel 1 × 1 filter, as shown in Figure 2. On the left side of the figure, there is a multiplication of an 8-bit input and a 4-bit weight. The 8-bit number is a fixed-point number that has been quantized by SCQ, which is the first step of M4bQ. On the right side of the image, the requantizing and adjusted calculations are displayed. The requantizing converts an 8-bit number into two 4-bit numbers, $f\_in\_odd_{4b}$ and $f\_in\_oven_{4b}$, and copies a weight. In other words, the re-quantizing converts the original single-channel input feature-map into two channels 1 × 1 and copies the weight into two channels as well. When performing convolution, multiply $f\_in\_odd_{4b}$ directly with a 4-bit weight, then multiply by 16 to obtain conv_odd, on the other hand, add 8 to $f\_in\_oven_{4b}$ first, and then multiply it by a 4-bit weight to obtain conv_even. Finally, add them together to obtain the result of adjusted convolution, which is the same as the calculation on the left side of the image.

The quantization formula for performing requantizing of M4bQ is shown in Equation (5), where $f\_in\_odd_{4b}$ and $f\_in\_even_{4b}$ represent the 4-bit quantized values, and $f\_in_{8b}$ is the quantized 8-bit value from SCQ. After quantization, the number of input channels doubles, necessitating that the weights of the first layer also be expanded by simply duplicating each channel into adjacent channels.

(5) $$\begin{array}{c} f\_in\_odd_{4b}=floor\left(f\_in_{8b}/16\right) \end{array}$$

(6) $$\begin{array}{c} f\_in\_even_{4b}=f\_in_{8b}\%16-8 \end{array}$$

The original 8b × 4b convolution of $f\_in_{8b}$ * weight can now be performed using two parity-based 4-bit convolutions, as shown in Equation (7).

(7) $$\begin{array}{c} f\_in_{8b}*weight=conv\_odd+conv\_even \end{array}$$

(8) $$\begin{array}{c} conv\_odd=f\_in\_odd_{4b}*weight\times 16 \end{array}$$

(9) $$\begin{array}{c} conv\_even=(f\_in\_even_{4b}+8)*weight \end{array}$$

The reason for adding the constant ‘8’ to Equations (5) and (7) is to limit the quantized number within the range of signed 4-bit, that is, −8 to 7. Without the constant 8, the value of $f\_in\_even_{4b}$ would exceed 7 and could no longer be stored as a signed 4-bit number, resulting in a mismatch with the 4-bit network input of the hardware.

Maintaining a consistent bit width for both the input activations and weights across all neural network layers will significantly reduce the complexity and enhance the efficiency of the convolution circuit. The trade-off is only a slight increase in memory footprint for weight storage in the first layer. Note that current quantization schemes, such as LSQ [24] or PArameterized Clipping acTivation (PACT) [26], generally achieve satisfactory performance when quantizing the weights of the YOLO model into 4 bits. Therefore, the proposed optimizations in our quantization scheme are only applied to input activations.

## 5. Accelerator Architecture

### 5.1. Overall Hardware Architecture

**Figure 3:**
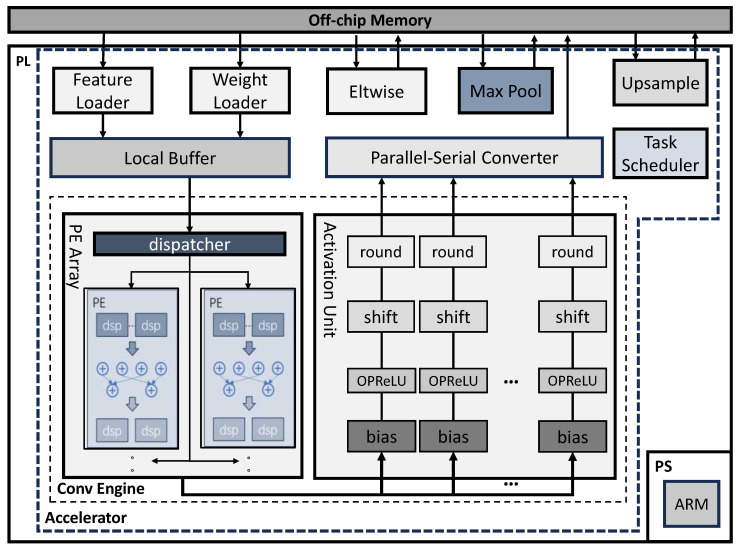
Overall structure of the proposed accelerator.

The top-level architecture of the proposed YOLO accelerator is shown in Figure 3.

An MPSoC FPGA device is used to deploy the accelerator. Computational-intensive layers of the YOLO model are accelerated on the Programmable Logic (PL) part, which includes a Processing Element (PE) Array for parallel convolution, a Local Buffer for caching feature-map and weight data, an Activation Unit for bias processing and result quantization, a central Task Scheduler for transmitting status data between different kernels and controlling the behavior of the system, and a group of function units for the eltwise, pooling and up-sample layers. The pre-processing and the post-processing procedures, which only account for a very small portion of the total workload of the YOLO model, are implemented in software on the ARM processor. During convolution, the feature-map and weights are first loaded into the Local Buffer and then sent to the PE Array. The intermediate result then enters the Activation Unit for bias addition, activations computation, and final quantization. This long pipelined processing mode can efficiently avoid frequent external memory access and guarantee the accelerator always works at the highest occupancy. Moreover, the whole YOLO network is processed in a layer-by-layer fashion to maximize hardware resource reuse.

### 5.2. Convolution Parallel Computing Data Flow

**Figure 4:**
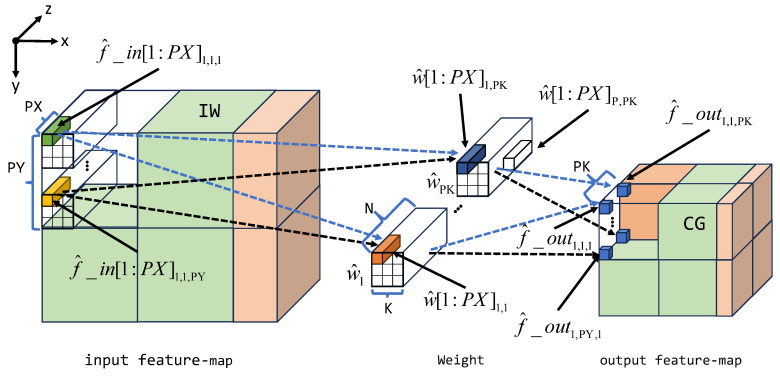
Convolution acceleration strategy.

In terms of data transporting and scheduling, as illustrated by Figure 4, the convolution of each layer is separated into multiple Convolution Groups (CG). Each CG is a sub-block in the output feature-map. Correspondingly, the entire input feature-map is divided into several Input Windows (IW) and the weights are divided into several groups of filters. The calculation of each CG will be carried out sequentially. As shown in Figure 3, the feature-map and weights will first be cached in the Local Buffer and then sent to the PE Array for parallel convolution calculation. After the calculation is completed, data will enter the Activation Unit in parallel and, finally, be written back to DDR in series. This chapter mainly introduces the parallel computing method of data in convolution calculation, and the specific implementation will be introduced later.

A pair of ping-pong buffers is designed inside the Local Buffer. Therefore, during the convolution process, the feature-map data of one CG can be fetched from the on-chip Local Buffer, while the data for the next CG can be loaded from external memory at the same time, hiding the memory access time between different CGs. The sliding window procedure of convolution is conducted along the *x*-dimension so that the weights stored in the Local Buffer can be reused within one CG to further minimize external bandwidth access.

Our accelerator is capable of performing parallel computation across three dimensions, i.e., input dimension *x* and output dimensions *y* and *z*. Within each dimension, distinct parallel computing methods are deployed to enhance processor performance. As shown in Figure 4, in order to calculate a CG, PK filters will participate in the convolution simultaneously, and each filter will be placed on PY different positions of the input feature-map and perform convolution calculations at each position simultaneously. After completing the convolution of this set of positions, the result of a slice in CG will be obtained. Afterward, each participating filter will move to the next set of PY positions, meaning that the PY convolution positions of each filter will move one step along the x-axis, and then perform convolution at the new position to obtain the result of the next slice in CG. In the calculation process of each filter at each position, vectors with a length of PV are multiplied and accumulated in parallel. Perform the above process until the entire CG calculation is completed, and start the calculation of the next CG.

To further explain, within the input dimension *x*, adjacent feature-map data are aggregated as a single vector data with a width of PX. Conversely, in the output dimensions *y* and *z*, parallel executions of PY and PK convolution tasks are undertaken, respectively. In each CG, a total number of PK kernels (i.e., convolution filters) are loaded by the accelerator, and each kernel is shared among PY convolutions along the input *y*-dimension. Each kernel is partitioned into P=K∗K∗N/PX weight vectors ${\hat{w}}_{p}$, where *K* denotes the kernel size, and *N* represents the channel depth. Subsequently, each weight kernel vector is multiplied by the corresponding input feature-map vector. The parallel computing design can be summarized by Equation (10), where $\hat{f}\_in\left[1:PX\right]$ and $\hat{w}\left[1:PX\right]$ denote the input feature-map and weight data vectors, and $\hat{f}\_out$ represents the convolution result. Each $\hat{f}\_out_{x,y,z}$ requires undergoing *P* times vectored dot product. Simultaneously, the accelerator executes a total of PY×PK parallel dot products, implying that PY×PK×PX Multiplier and Accumulation (MAC) operations are conducted at every cycle.

(10) $$\hat{f}\_out_{x,y,z}=\sum_{p=1}^{P}\hat{f}\_in{\left[1:PX\right]}_{p,x,y}\cdot \hat{w}{\left[1:PX\right]}_{p,z}$$

### 5.3. Local Buffer

The Local Buffer in Figure 3 is designed to cache the data required for calculating each CG in Figure 4. Its internal ping-pong buffer can support reading the data required for the next CG from the Feature Loader and Weight Loader in Figure 3 while sending the data required for the current CG to the PE Array in Figure 3, thereby hiding the data transport time.

**Figure 5:**
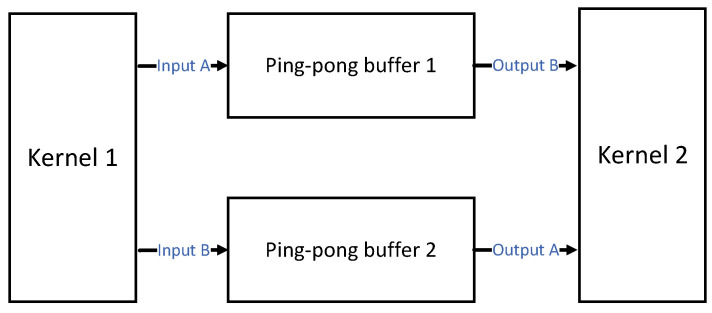
The ping-pong buffering strategy used in the design of the Local Buffer unit.

The ping-pong operation is a widely used data flow control technique that can act as a buffer between FPGA kernels, ensuring smooth data flow between upstream and downstream. For example, as shown in Figure 5, the ping-pong operation is used to transfer data between kernel 1 and kernel 2. In stage A, kernel 1 will write data to ping-pong buffer 1, while kernel 2 will read data from ping-pong buffer 2. In stage B, kernel 1 will write data to ping-pong buffer 2, while kernel 2 will read data from ping-pong buffer 1. Alternating between stages A and B completes uninterrupted asynchronous read and write between kernel 1 and kernel 2.

**Figure 6:**
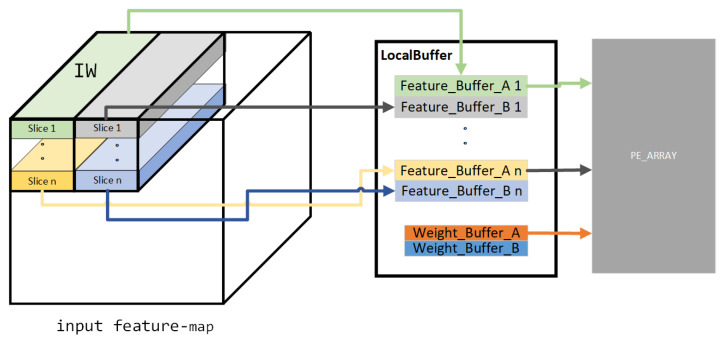
Detailed design of the Local Buffer.

In the proposed accelerator, ping-pong operations are applied in the Local Buffer to achieve smooth data flow. The Local Buffer stores the feature-maps and weights data required for the two adjacent CGs mentioned in Figure 4. Each IW is stored as a slice, and each filter is stored together. The Local Buffer is scheduled by the Task Scheduler in Figure 3 and will respond to input information in various ways. As shown in Figure 6, the data of two adjacent IWs in Figure 4 will be stored in two sets of ping-pong buffers, namely Feature_Buffer_A 1 to Feature_Buffer_A n and Feature_Buffer_B 1 to Feature_Buffer_B n. The two adjacent sets of weight data will be stored in two ping-pong buffers, Weight_Buffer_A and Weight_Buffer_B. At the initial startup of this system, there is no data in the Local Buffer. The Feature Loader and Weight Loader in Figure 3 will read the first IW and corresponding weights required for the first CG and store them in the Feature_Buffer_A 1 to Feature_Buffer_A n and Weight_Buffer_A. Afterward, the PE matrix will initiate the calculation process, gradually reading data from the “A” buffers group for the calculation of the first CG. At the same time, the Feature Loader and Weight Loader will read the data of the second CG and enter it into the Feature_Buffer_B 1 to Feature_Buffer_B n and Weight_Buffer_B. Until the first CG has been calculated and all the data for the second CG have been read into the Local Buffer, the PE array will gradually retrieve the data for the second CG. At this point, the Feature Loader and Weight Loader will read the IW and weights required for the third CG. This read/write process continues until the entire layer is computed, after which the convolution of the next layer is initiated.

More importantly, the sliced storage strategy for feature-maps in the Local Buffer can provide parallel feature-map reading for the PE Array. During computation, data can be simultaneously read from each Feature_Buffer to provide parallel feature-map data for the PY parallel computed convolution positions in Figure 4.

### 5.4. PE Design

**Figure 7:**
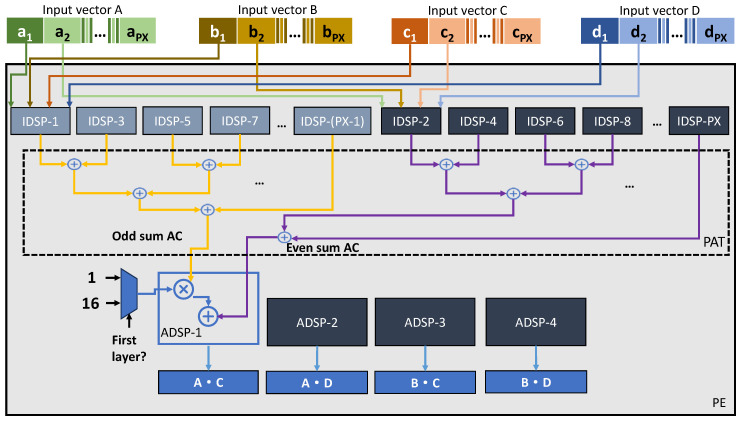
Architecture of PE.

Processing Element (PE) is the basic unit of the PE Array in Figure 3. After receiving data from the Local Buffer, the dispatcher in Figure 3 converts them into multiple vector pairs and sends them to the PEs. Each PE processes 4 vector calculations at the same time. Then, the result of each PE will be sent to the Activation Unit for parallel bias, activation, and quantization, and finally sent back to DDR serially through Parallel–Serial Converter. The internal structure of the PE in the PE Array is shown in Figure 7.

In the proposed accelerator, (PY/2)∗(PK/2) PEs run in parallel to achieve the parallel convolution design in Figure 4. Each PE is responsible for generating 4 adjacent results in a slice of CG, namely $\hat{f}\_out_{x,y,z}$, $\hat{f}\_out_{x,y+1,z}$, $\hat{f}\_out_{x,y,z+1}$, and $\hat{f}\_out_{x,y+1,z+1}$. To achieve this, the PE will perform K∗K∗N/PX calculations and each calculation contains 4 inner products of the vector of length PX. To be more specific, at each calculation, the PE receives 4 vectors, namely *A*, *B*, *C*, and *D*, then outputs the A·C, A·D, B·C, and B·D as the result.

Each PE consists of a number of PX Input DSPs (IDSP) at the top, a group of Parity Adder Trees (PAT) in the middle, and 4 additional Addition DSPs (ADSP) at the bottom. The IDSPs are configured in the proposed DSP packing mode to support parallel 4-bit multiplications as described in the previous section, that is, each IDSP computes 4 scalar multiplications. For example, IDSP-i takes $a_{i}$, $b_{i}$, $c_{i}$, and $d_{i}$ as input, and the output is $a_{i}*c_{i}$, $a_{i}*d_{i}$, $b_{i}*c_{i}$, and $b_{i}*d_{i}$. The output of each IDSP will be collected by a PAT below. There are a total of 8 PATs, each responsible for computing OddsumAC, EvensumAC, OddsumAD, EvensumAD, OddsumBC, EvensumBC, OddsumBD, EvensumBD. Figure 7 shows two PATs that collect scalar results of $a_{i}*c_{i}$ from IDSPs with odd numbers and even numbers, respectively, and finally sum them to obtain OddsumAC and EvensumAC. It is proposed to support the parity convolutions as defined by Equation (7), which requires different operations for channels in odd positions and channels in even positions. Then, the results of the PATs are sent to the ADSPs and the calculation of PE is performed.

**Figure 8:**
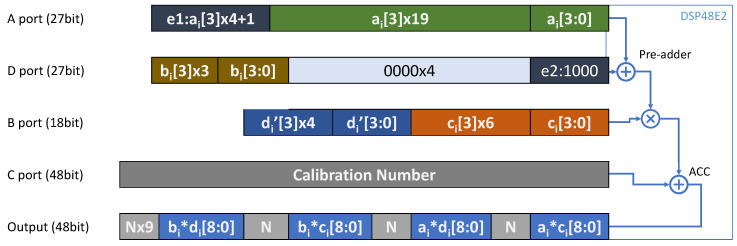
DSP packing scheme.

If the calculation of PE is the convolution of the first layer quantized by M4bQ, the constant offset values of 8 for the channels in even positions are added to the input data by setting the e1 and e2 in the DSP block, as shown by Figure 8. Also, the constant scale value of 16 is multiplied by the odd partial results by using the ADSPs. As a result, the computation of Equation (7) is implemented. For normal convolutions, the e1 and e2 are not set in DSP packing and scale values in ADSPs are set to 1. In this way, the PE array can be reused for M4bQ and normal quantization without any functional modification. The PE design proposed in this paper cleverly utilizes the pre-adder, post-adder, and multiplier in the DSP block to implement various convolution computations. Additionally, it supports the first layer quantization algorithm proposed in this paper without requiring additional hardware resources, achieving a unified 4-bit convolution computation for the entire YOLO network.

### 5.5. DSP Packing Scheme

The proposed DSP Packing Scheme is used in IDSP in Figure 7. By using the proposed 4-bit quantization scheme, the feature-map and weight data are quantized with reduced bit width, thus more MAC operations can be packed and executed by a single DSP block, resulting in improved throughput of the hardware accelerator.

The DSP packing proposed in this article, as shown in Figure 8, can enable four 4-bit multiplications to be completed in one DSP calculation, and supports first-layer M4bQ and ordinary 4-bit quantization in the rest of the network. Figure 8 shows the packing scheme on DSP48E2, and by removing the two leftmost bits at ports A and D, this scheme can also be applied on DSP48E1. $a_{i}$, $b_{i}$, $c_{i}$, and $d_{i}$ (i∈[1:PX]) represent the input operands of the 4 MAC operations $b_{i}*d_{i}$, $b_{i}*c_{i}$, $a_{i}*d_{i}$, and $a_{i}*c_{i}$ performed by DSP packing. These 4 input operands come from the parallel computed vector with a length of PX in Figure 4. Among them, $a_{i}$ and $b_{i}$ are 4-bit signed numbers, representing two scalar elements of the input feature vector of $\hat{f}\_in{\left[1:PX\right]}_{p,x,y}$ and $\hat{f}\_in{\left[1:PX\right]}_{p,x,y+1}$ in adjacent positions, respectively. $c_{i}$ and $d_{i}$ are 4-bit signed numbers, representing two scalar elements of $\hat{w}{\left[1:PX\right]}_{p,z}$ and $\hat{w}{\left[1:PX\right]}_{p,z+1}$.

**Figure 9:**
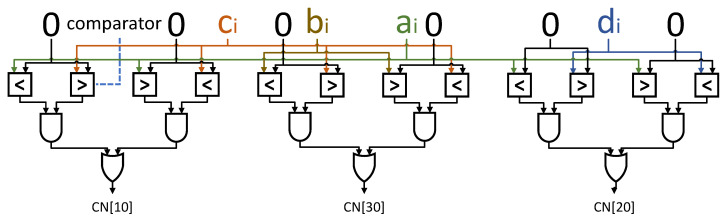
Circuit for Correction Number.

The way to package DSP is as follows: first, assign $c_{i}$ to the B port and $a_{i}$ to the A port. Afterward, place the $b_{i}$ sticker on the left side of the D port and set the remaining bits to 0. Then, add $\left(d_{i}\ll 10\right)$ to $c_{i}$, and place the result on the left side of port B, which is the $d_{i}^{\prime }$. Finally, set the Correction Number (CN). Due to the influence of sign bits of low-position results, high-bit data may be disturbed. For example, the sign bits of $a_{i}*c_{i}$ may cause a decrease in $a_{i}*d_{i}$. Because this result is actually equivalent to $a_{i}*c_{i}+\left(a_{i}*d_{i}\ll 10\right)$, $a_{i}*d_{i}$ will be affected by the sign bits of $a_{i}*c_{i}$. Therefore, a CN is placed in the C port. The bits CN[10], CN[20] and CN[30] are generated by using the circuit shown in Figure 9, while the rest of the bits are set to zeros. This completes the packaging of regular 4-bit multiplication.

Additionally, the e1 and e2 are optional parameters that are only used in M4bQ of the first layer, which offer two constants ’8’ for $a_{i}$ and $b_{i}$ according to the $f\_in\_even_{4b}+8$ in Equation (7). For e2, only set the lower four bits of port D to be 1000. For e1, the 4 highest bits of port A, need to be taken out and added by 1. The result is equivalent to $a_{i}+\left(8\ll 20\right)$.

In this way, the convolution operations for $\hat{f}\_out_{x,y,z}$, $\hat{f}\_out_{x,y+1,z}$, $\hat{f}\_out_{x,y,z+1}$ and $\hat{f}\_out_{x,y+1,z+1}$ can be implemented by PX DSP blocks in parallel, which will be discussed in the next chapter. After computing, take the result from the Output port. The *N* as a placeholder in Figure 8 is meaningless.

It is worth noting that the DSP packing scheme proposed in this paper is different from [16], mainly reflected in three aspects: Firstly, the DSP packing scheme promoted in [16] is based on the assumption that activations are unsigned numbers and weights are signed numbers, while in our scheme, both activations and weights are signed numbers. Second, our correction number is calculated in advance and does not require reading the DSP’s results, allowing pipelined datapath to run with II = 1 to maximize throughput. Lastly, the paper [16] does not consider the 4-bit input quantization of the first layer, while the proposed DSP packing scheme supports special calculations required for the M4bQ (our proposed input quantization method of the first layer).

## 6. Bandwidth Optimization

In order to adapt to 4-bit quantization, we optimized the data access of external DDR memory. The trivial idea is to store 4-bit data in an 8-bit char.

**Figure 10:**
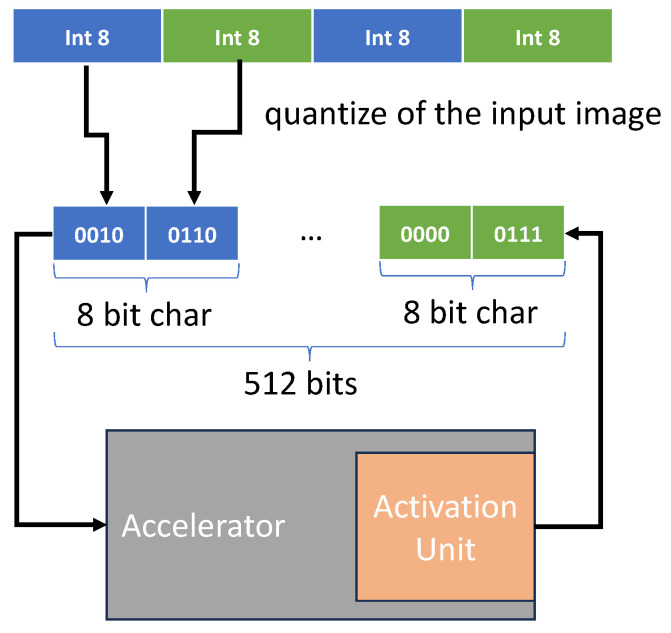
DDR storage optimization.

However, the above method did not utilize the advantages of 4-bit quantization, so we use the concatenation storage as shown in Figure 10. After the input image is quantized to 4 bits by M4bQ, two adjacent 4-bit numbers are concatenated and stored in an 8-bit char. Then, multiple 8-bit chars are further grouped into a 512-bit word, which matches the width of the data bus of the DDR memory controller. When the FPGA accelerator interacts with DDR, the burst method is used, which continuously reads or writes multiple groups of 512 bits of data. In this way, the number of DDR accesses can be reduced by half and the utilization of bandwidth is more efficient.

The quantization and 4-bit concatenation of the input image of the first layer is completed in the Processing System (PS), and the rest of the quantization and 4-bit concatenation is performed by PL, more specifically, in the Activation Unit of the Figure 3. Therefore, due to the fact that the input 4-bit quantization and concatenation completed by the PS part only needs to be executed once during image processing, while the remaining quantization and concatenation are performed through a pipeline in the PL part, it will hardly have a negative impact on the inference latency.

Based on the experiments, the average external memory bandwidth usage of the implemented accelerator is 6.10 GB/s, which is much lower than the bandwidth limit of 12.8 GB/s of DDR4 memory.

## 7. Experiment Results

### 7.1. Experimental Environments

**Figure 11:**
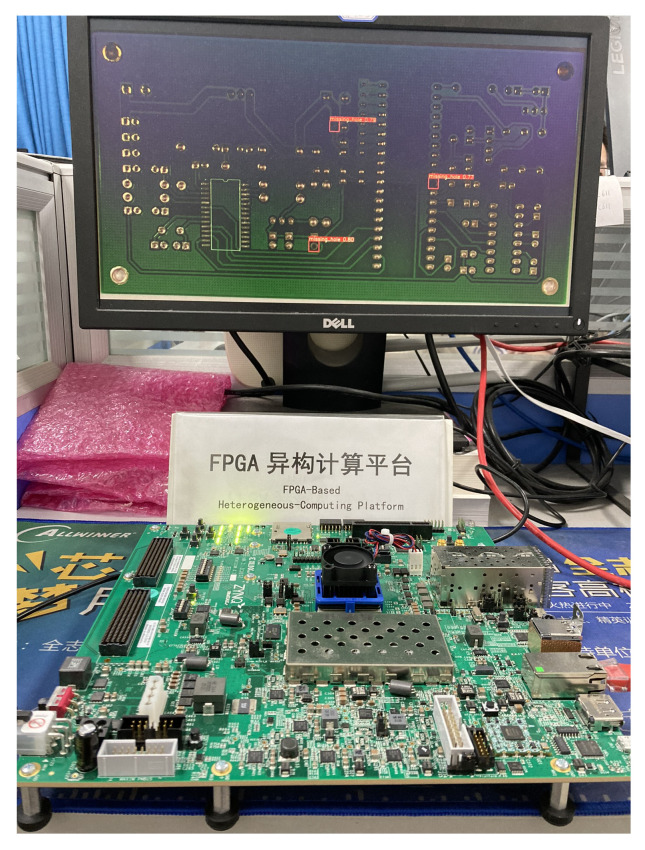
ZCU102 board.

In this paper, YOLOv5s was mapped onto the proposed accelerator to evaluate performance and efficiency. Due to the different performances of industrial inspection equipment in various fields and datasets, in order to fairly evaluate and compare the proposed quantization algorithm, we compared the model accuracy in the VOC dataset with other FPGA accelerators, which is detailed in Section 7.4. Simultaneously, we deployed a PCB defect detection model for industrial applications, which is detailed in Section 7.3. The model quantization training process was conducted on the VOC Dataset(2007 + 2012) and PKU-Market-PCB using PyTorch (version 1.13.1) with a size of 512 × 512. The proposed accelerator was implemented using Xilinx’s HLS flow (version 2022.1) and deployed on the ZCU102 board as shown in Figure 11, which features a Zynq UltraScale+ MPSoC with 4 GB DDR4 memory. The MPSoC device provides 2520 DSP blocks, 32.1 Mb block RAM, and 33,958 CLB resources.

### 7.2. Dataset

#### 7.2.1. VOC Dataset

**Figure 12:**
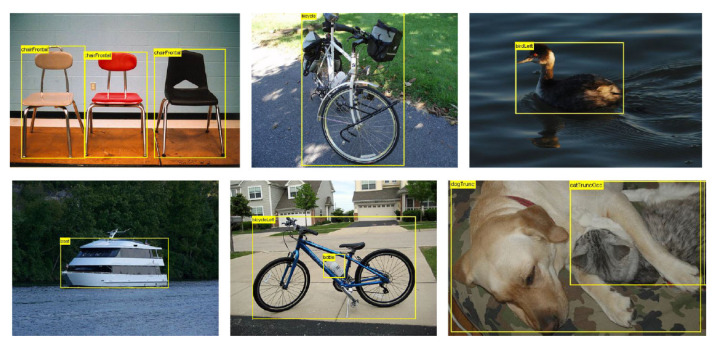
Sample images from the VOC dataset [27].

The PASCAL Visual Object Classes (VOC) Challenge [27] is a world-class computer vision challenge. The organizer provided rich images with 20 types of annotations for the object detection task and defined a standard method for model accuracy evaluation. It has been recognized as the benchmark for object detection. Figure 12 shows a sample image from the dataset.

#### 7.2.2. PKU-Market-PCB

**Figure 13:**
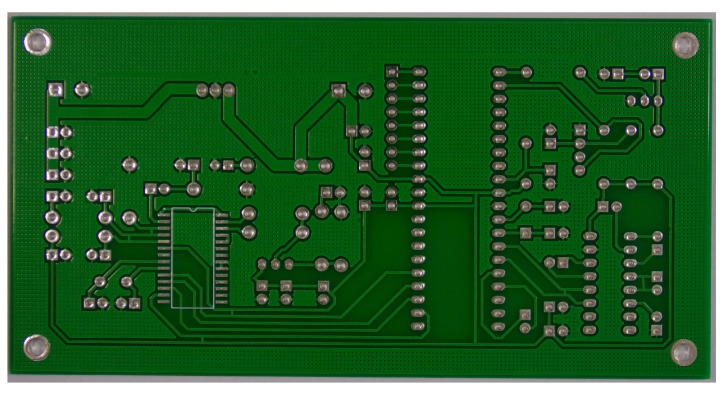
Sample images from the PCB dataset.

The PKU-Market-PCB [28] is a public dataset for detecting PCB defection, which includes 1386 images and annotations for six types of defects (missing holes, mouse bites, spur, short circuits, open circuits, spurious copper). It can be used for detection, classification, and registration tasks in the PCB board preparation process. Figure 13 shows a sample image from the dataset.

### 7.3. PCB Defect Detection

**Figure 14:**
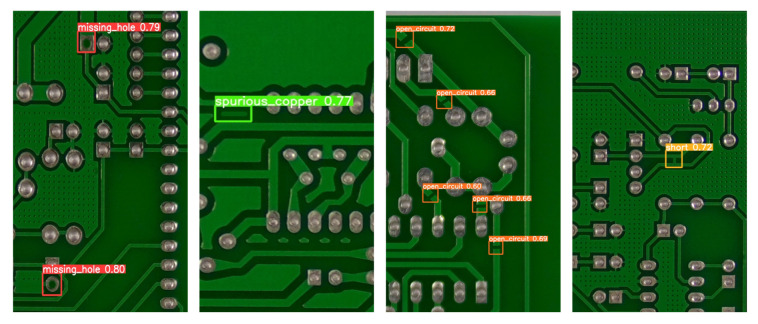
Detection results of PCB defect.

In order to detect PCB defects, this paper first trains a full precision model on the PCB dataset, then quantizes the model using the proposed scheme, and finally deploys the proposed accelerator for inference on ZCU102, constructing a PCB defect detection system with a detection speed of 79.1 FPS with an image size of 512 × 512. The detection result is shown in Figure 14. It can be proven that the quantization and hardware design proposed in this article are effective for industrial datasets and have high application value.

### 7.4. Analysis and Comparison

The hardware resource breakdown of the final design is summarized in Table 1. The main computing kernel, referred to as the Conv Engine, consumes the majority of the logic and DSP resources. Specifically, a total of 1600 DSP blocks are utilized, supporting 4096 parallel 4-bit multiplications per cycle during convolution. Consequently, the theoretical peak performance of the accelerator is 1638.4 GOP/s at a frequency of 200 MHz. For YOLOv5s, the measured frame rate was 79.1 FPS, delivering a throughput of 808.6 GOPS and 0.49 GOPS/DSP with an input size of 512 × 512 and a power consumption of 12.3 W.

Table 2 compares the performance and resource consumption of the proposed design with several state-of-the-art FPGA-based YOLO accelerators. As the designs are implemented on different FPGA platforms, we use DSP efficiency (GOPS/DSP) and GOPS as metrics to objectively evaluate the performance advantages of each approach.

Compared with the traditional 16/8-bit designs [5,30], the proposed accelerator demonstrates 106% throughput improvements for the YOLOv5s model and 28.9% GOPS/DSP improvements for the YOLOv3 model without considering the 40% higher working frequency used by [30] and 3.7 times logic resources consumed by [5]. Notably, ref. [30] is a commercial FPGA accelerator design based on highly optimized RTL codes. Additionally, the proposed design consistently shows more than 2× DSP efficiency over [5]. Compared with the latest low-bit-width designs of [12,29], which are implemented on different FPGA devices, our approach shows approximately 107% advantages in GOPS. Compared with FPGA accelerators used in industrial applications [31], our accelerator has significant advantages more than 10 times in both GOPS and GOPS/DSP.

Our design maintains an inference accuracy drop of around 1.5% mAP for YOLOv5s models, which is significantly lower than [12] (3%) and [29] (2.9%). This minimal accuracy degradation showcases the effectiveness of our quantization scheme in preserving model accuracy while reducing implementation costs. From the above comparison, it is evident that the proposed YOLOv5 hardware accelerator design demonstrates remarkable advantages in both performance and resource efficiency over previous studies.

In addition, compared with the accelerator based on Tensor Train Decomposition (TT) [32], our accelerator has a comparable level of mAP reduction, but with higher hardware utilization, especially with DSP utilization (GOPS/DSP) as high as 15.3 times. On the other hand, compared with accelerators [15] that use LUTs as the main computing device, the accelerator in this article does not have an advantage in hardware efficiency, but whether it can achieve 800 GOPS while still maintaining the same hardware efficiency remains to be experimentally verified.

In the comparison against other devices presented in Table 3, our FPGA accelerator exhibits a nearly 4× advantage in throughput (GOPS) and a remarkable 61-fold enhancement in energy efficiency compared with the CPU. When compared with the GPU implementations, our accelerator maintains its superiority, yielding a nearly 5× improvement in energy efficiency.

## 8. Conclusions

We have introduced several quantization and accelerator optimization methods to achieve an efficient FPGA-based accelerator. On the quantization front, we developed Mimic 4-bit Quantization (M4bQ), Offset PReLU (OPReLU), and Signed Clipping Quantization (SCQ), which together compress the model to 4 bits with only a 1.5% reduction in accuracy. For hardware optimization, we proposed an efficient DSP packing strategy and a resource-efficient, flexible Processing Element (PE) design, which significantly enhances throughput and DSP utilization. The proposed quantization and accelerator designs are versatile and can be adapted to various convolutional neural networks. To meet the stringent requirements for Printed Circuit Board (PCB) defect detection, particularly in terms of model accuracy and inference speed, we selected YOLOv5 for implementation. Experimental results demonstrate that our accelerator meets the real-time processing and accuracy demands of industrial visual applications.

## Figures and Tables

**Table 1:** Hardware resource utilization of each unit.

Kernel Name	LUT	Memory (BRAM)	DSP
Upsample	3475 (2.2%)	4 (0.7%)	18 (1.0%)
Weight Loader	3302 (2.1%)	7.5 (1.3%)	1 (0.0%)
Parallel–Serial Converter	7485 (4.7%)	2 (0.4%)	6 (0.4%)
Feature Loader	2749 (1.7%)	2 (0.4%)	16 (1.0%)
Local Buffer	11,526 (7.2%)	399 (71.5%)	0 (0.0%)
Max Pool	5354 (3.3%)	8 (1.4%)	0 (0.0%)
Eltwise	5516 (3.4%)	6 (1.1%)	0 (0.0%)
Task Scheduler	861 (0.5%)	0 (0.0%)	6 (0.4%)
Conv Engine	105,698 (66.0%)	2 (0.4%)	1600 (97.1%)
Others ^1^	14,083 (8.8%)	127.5 (22.8%)	0 (0.0%)
Total	160,049 (100.0%)	558 (100.0%)	1647 (100.0%)

^1^ The ‘Others’ refers to the hardware resources that are consumed by the infrastructure IPs, including DDR memory controller, AXI interconnectors, etc.

**Table 2:** Comparison with previous works.

	[5]	[12]	[29]	[30]	[31]	[15]	[32]	Ours
Year	2023	2024	2021	2020	2022	2023	2021	2024
Model	YOLOv5s	YOLOv4-tiny	YOLOv3	YOLOv3	YOLOv4-tiny	YOLOv2	YOLOv5s	YOLOv5s
Dataset	COCO	COCO	COCO	VOC	Coal Gangue	none	VOC	VOC
Quantization	A16W8	A8W5	A4W4	A8W8	A16W16	A4W4	TT ^1^	A4W4
mAP(%)	56.2 (none)%	79 (−3)%	53.9 (−2.9)%	75.3 (±0)%	96.35 (−1.25)%	none	76.9 (−1.5)%	73.8 (−1.5)%
Input Size	640	416	320	416	416	416	640	512
Platform	VCU110	ZC706	XC7Z045	ZCU102	ZYNQ-7020	ZU9EV	KCU116	ZCU102
Frequency	200	200	100	281	none	250	none	200
BRAM(36k)	1888	525	255.5	765	96	86.5	220	558
DSP	1794	640	900	2130	216	21	1321	1647
LUT	602k	172k	145k	156.5k	41.9k	27k	182k	160k
GOPS	392.0	390.6	390	816.4	9.24	140.3	42.6	808.6
GOPS/DSP	0.22	0.61	0.43	0.38	0.04	6.70	0.03	0.49

^1^ TT means Tensor Train Decomposition, which is a model compression method that differs from quantization.

**Table 3:** Comparison with CPU and GPU.

Device	Quantization	Inference Time (ms)	GOPS	Power (W)	GOPS/W
FPGA ZCU102 (our)	4 bit	12.633	808.6	12.3	65.7
CPU E5-2686 v4 [18]	float32	98	168.4	160	1.1
GPU NVIDIA® V100 [18]	float32	6.4	2578.1	250	10.3

## Data Availability

The original contributions presented in the study are included in the article, further inquiries can be directed to the corresponding author.
